# Prevalence and Endoscopic Documentation of *Helicobacter pylori* and Gastric Precancerous Conditions in Routine Gastroscopy with Biopsies: A Cross-Sectional Study

**DOI:** 10.1055/a-2992-1623

**Published:** 2026-11-06

**Authors:** David Andreas Roser, Hanna Ganthaler, Andreas Probst, Yevgeniia Ignatenko, Dennis Freuer, Helmut Messmann, Christoph Römmele

**Affiliations:** 1 Department of Gastroenterology 39694 University Hospital AugsburgAugsburgBavariaGermany; 2 Medical Faculty 39694 University Hospital AugsburgAugsburgBavariaGermany; 3 Institute of Digital Medicine 39694 University Hospital AugsburgAugsburgBavariaGermany; 4 Institute of Epidemiology 39694 University Hospital AugsburgAugsburgBavariaGermany

**Keywords:** precancerous conditions & cancerous lesions (dysplasia and cancer) stomach, diagnosis and imaging (inc chromoendoscopy, NBI, iSCAN, FICE, CLE), image and data processing, documentation, quality management, performance and complications

## Abstract

**Background:**

In Western countries without screening programs, *Helicobacter pylori* infection, gastric atrophy, and intestinal metaplasia must be detected opportunistically during endoscopy, yet real-world data on how reliably this occurs are scarce. We determined their histologic prevalence among biopsied diagnostic gastroscopies and how completely they are recorded in routine endoscopy reports.

**Materials and Methods:**

Retrospective single-center cross-sectional study of all diagnostic gastroscopies with gastric biopsies at a tertiary university hospital (2016–2025). Documentation of each lesion was compared with institutional histopathology reports. As only biopsied examinations were eligible, concordance measures reporting completeness. Prevalence was analyzed at patient level ( *n* = 9,852) and concordance at examination level ( *n* = 11,381).

**Results:**

*H. pylori* infection or any gastric precancerous condition was present in 31.4% of patients ( *H. pylori* 11.5%, atrophy 6.6%, intestinal metaplasia 21.5%). A corresponding finding was recorded in the endoscopy report in 9.6% (95% CI 8.0–11.4) of *H. pylori* -positive, 9.2% (8.1–10.3) of metaplasia-positive and 49.4% (45.8–52.9) of atrophy-positive examinations. Counting rapid urease tests, which indicate *H. pylori* testing but not written mucosal suspicion, increased documentation to 46.0%. Results were similar with antrum-and-corpus sampling (90.7%; ≤0.7 percentage points). The publication of MAPS-II or newer endoscopy generations did not increase documentation substantially.

**Conclusion:**

*H. pylori* infection and gastric precancerous conditions affected nearly one-third of biopsied patients, yet most histologically confirmed findings were not explicitly documented at endoscopy. Closing this documentation gap should be a priority for integrating gastric cancer risk assessment into routine endoscopic reporting.

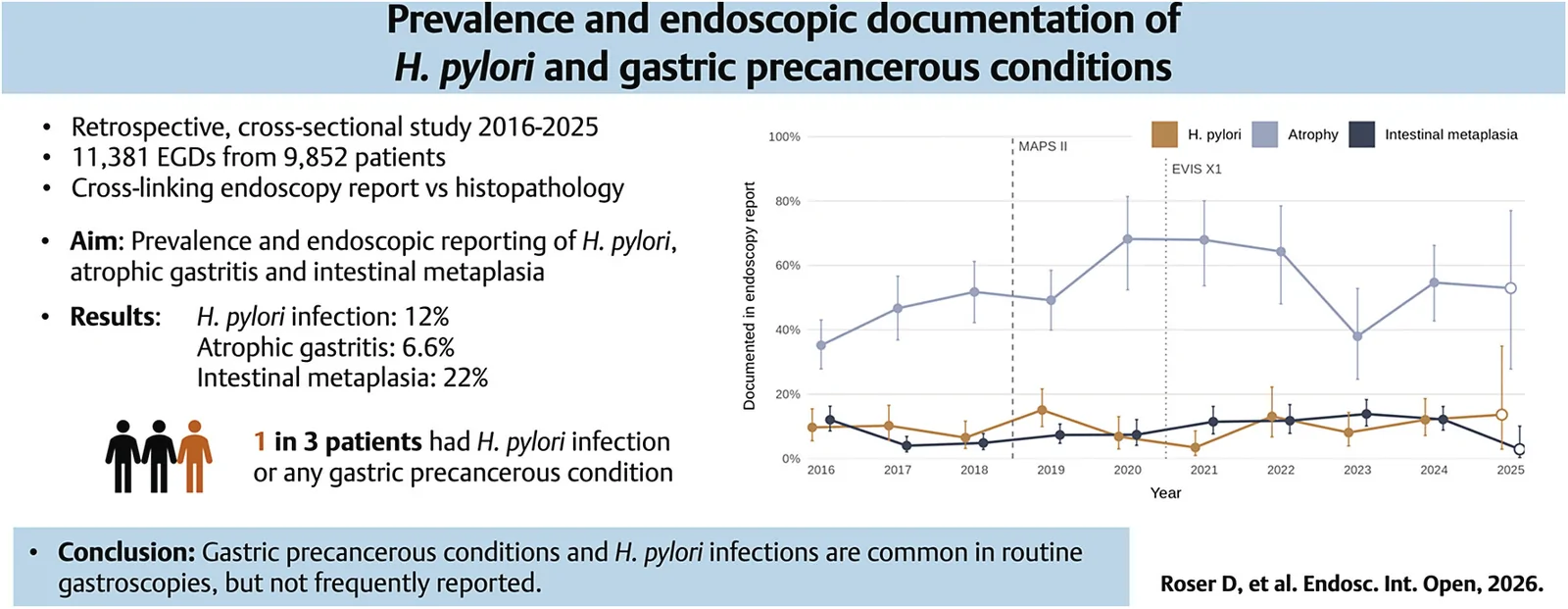

## Introduction

Gastric cancer remains one of the leading causes of cancer-related death in Europe, 1 even though its biological progression is among the best characterized of all solid tumors. Chronic *Helicobacter pylori* infection, classified as a Group 1 carcinogen by the World Health Organization, 2 drives the Correa cascade through atrophic gastritis and intestinal metaplasia (IM) toward dysplasia and adenocarcinoma over years to decades. 3 This trajectory offers a long window of opportunity for prevention through detection, eradication therapy, and risk-adapted surveillance. The clinical implications of these findings differ in scope and severity: *H. pylori* identification routinely triggers eradication therapy, 4 whereas atrophy and IM guide risk-adapted surveillance when extensive or histologically advanced, for example, when reaching OLGA/OLGIM stage III–IV or an EGGIM score of ≥5 according to current MAPS-III guidelines. 5

The technical prerequisites for opportunistic detection are widely in place across Europe. High-definition endoscopy and image-enhancement technologies have markedly improved the endoscopic visibility of mucosal patterns associated with precancerous changes, 6 and structured frameworks such as OLGA/OLGIM, EGGIM and the Kimura–Takemoto classification (KTC) provide validated tools for histologic and endoscopic risk stratification. The Kyoto classification additionally provides standardized endoscopic features for assessing *H. pylori* infection status, distinguishing current infection, past infection, and noninfection. 7 In controlled studies with expert endoscopists, endoscopic assessment achieves high accuracy for the detection of IM and reliable phenotypic grading of atrophic changes. 8

Despite this favorable combination of biological understanding, technical capability, and explicit guideline recommendations, gastric cancer continues to be predominantly diagnosed at advanced stages in Western countries. In Germany, only a minority of cases are identified at an early, curatively treatable stage. 9 Population-based upper gastrointestinal screening is not routinely implemented in low-to-intermediate incidence settings, leaving opportunistic identification of *H. pylori* and gastric precancerous conditions during diagnostic esophagogastroduodenoscopy (EGD) performed for other indications as the main preventive lever. 5 10 It is unclear, however, to what extent the capabilities demonstrated under controlled study conditions translate into routine clinical care. Real-world data on the histologic prevalence of these conditions and on their endoscopic documentation outside dedicated study settings remain limited, particularly from large unselected Western cohorts.

To address this gap, we conducted a retrospective analysis of all diagnostic upper gastrointestinal endoscopies with gastric biopsies over a 10-year period. We hypothesized that (1) histologically confirmed gastric precancerous conditions are common in this routine tertiary-care population, with the prevalence of atrophy and IM increasing with age and (2) routine endoscopic documentation records only a fraction of these histologically confirmed lesions, reflecting a substantial gap between endoscopic capability under expert study conditions and documentation in standard care. Accordingly, the study had two co-primary aims: (1) to characterize the histologically confirmed prevalence of *H. pylori* infection, atrophic gastritis, and IM among examinations in which gastric biopsies were obtained and (2) to quantify the concordance between routine endoscopic documentation and institutional histopathology reports as the reference standard.

## Materials and Methods

### Study Design

We conducted a retrospective single-center cross-sectional study using routinely collected health data at University Hospital Augsburg, Germany, including all diagnostic upper gastrointestinal endoscopies with gastric biopsies performed between January 1, 2016 and March 31, 2025. The study was approved by the Ethics Committee of the University of Regensburg (approval no. 24-3812-104), which was the responsible ethics committee at the time of approval, in accordance with the Declaration of Helsinki and is reported in accordance with the Strengthening the Reporting of Observational Studies in Epidemiology (STROBE) statement.

### Study Population and Data Sources

We included all diagnostic EGDs with at least one gastric biopsy in adults (≥18 years). We excluded therapeutic upper endoscopies, as these represent a preselected, neoplasia- or complication-enriched population; non-EGD procedures of the upper or proximal gastrointestinal tract; examinations without gastric biopsies or without conclusive histopathology reports; and duplicate records.

Owing to a hospital information system transition in 2022, data were extracted in two separate extractions (January 2016–June 2022 and July 2022–March 2025). In both, only examinations with linked gastric biopsies were retrieved. Jejunoscopies and therapeutic procedures could be filtered automatically from July 2022 onward only and were excluded by manual review for the earlier period ( Fig. 1 ).

**Fig. 1:**
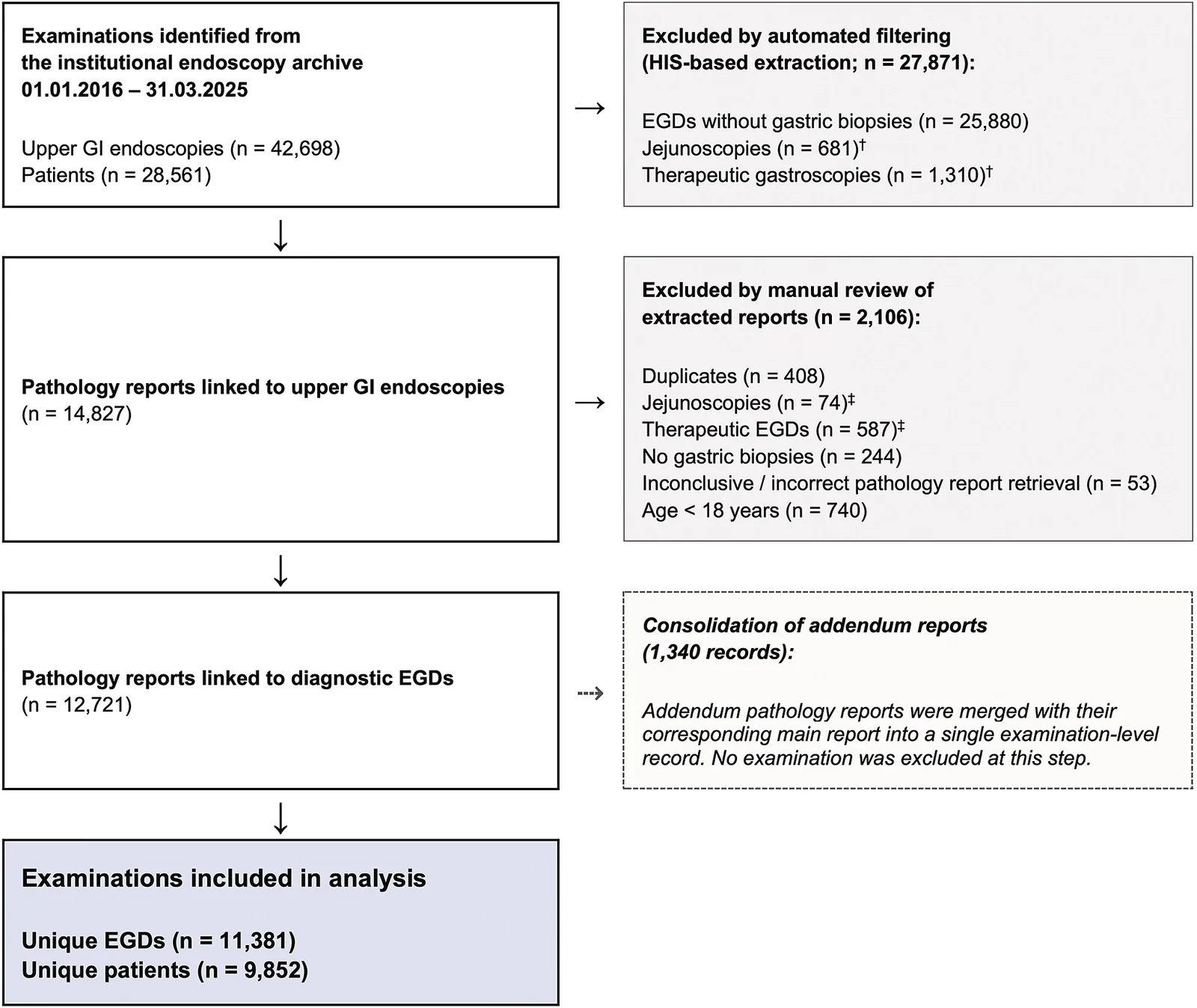
Study flow diagram. Examinations were extracted via the Medical Data Integration Center in two separate extractions (January 2016–June 2022 and July 2022–March 2025) owing to a transition of the hospital information system (HIS) in 2022. Identical inclusion and exclusion criteria were applied to both periods. Only examinations with linked gastric biopsies were extracted in both periods (upper exclusion box). † Jejunoscopies and therapeutic procedures could be filtered automatically only for examinations from July 2022 onward. ‡ For January 2016–June 2022, they were identified and excluded during manual review of the extracted reports (lower exclusion box). Counts refer to pathology report records until consolidation of addendum reports and to examinations thereafter. Patient-level counts in the final box represent unique individuals. EGD = esophagogastroduodenoscopy; HIS = hospital information system.

### Outcomes and Variables

#### Primary and Secondary Outcomes

This study had two co-primary outcomes: (1) the histologically confirmed prevalence of *H. pylori* infection, atrophic gastritis, and IM at the patient-level index examination; and (2) the concordance between routine endoscopic documentation and institutional histopathology reports as the reference standard, quantified separately for each of the three lesions. Because only biopsied examinations were eligible, outcome (2) quantifies the endoscopic reporting completeness. For *H. pylori* , this specifically measures agreement between documented endoscopic suspicion and histologic detection, not the adequacy of clinical testing.

Secondary outcomes were the composite outcome “ *H. pylori* infection or any gastric precancerous condition” (presence of at least one of *H. pylori infection* , atrophic gastritis, or IM); associations of prevalence with age group and sex; and the reported anatomical distribution and histologic extent of atrophy and IM.

#### Variables and Data Extraction

The extracted dataset comprised, for each examination, age at examination, sex, examination date, the full free-text endoscopy report, the gastric biopsy site(s) as documented by the endoscopist, and the corresponding histopathology report including any addendum reports linked at the examination level.

#### Histologic Outcomes

Target lesions were *H. pylori* infection, gastric atrophy/atrophic gastritis (atrophy), and IM extracted from the original pathology reports and coded as binary labels (present/absent) per examination. Addendum reports were considered where available. An examination was labeled positive if at least one biopsy from a given site was positive. Staining methods, including immunohistochemistry, were not standardized across the study period and were not analyzed. Explicit OLGA/OLGIM staging was assessed by searching the original pathology reports. Anatomical involvement (antrum, corpus, fundus) was recorded for atrophy and IM. IM was additionally classified at the patient level as distal (antrum only) or extensive (any corpus and/or fundus involvement). Because antral atrophy is difficult to distinguish histopathologically from chemically or reactively induced alterations (such as type C gastritis), it is prone to underreporting. 11 Atrophy is therefore reported as overlapping single-site categories, and the histologic reference standard for atrophy effectively captures corpus-predominant atrophy. Atrophy was recorded only when explicitly reported and was not inferred from IM. Dysplasia and gastric neoplasia were outside the scope of the approved study protocol and were not systematically extracted.

#### Endoscopic Documentation

Endoscopic suspicion of *H. pylori* , atrophy, and IM was extracted from the routine endoscopy reports and coded as binary variables, with a positive label assigned whenever the performing endoscopist documented either a definitive diagnosis or a written suspicion of the respective lesion. This inclusive definition reflects the clinical reality that any endoscopic suggestion typically prompts biopsy sampling. Rapid urease testing (RUT) was used during the study period but was not systematically captured by the original endoscopic suspicion label. A post hoc analysis assessed documented RUT performance in the biopsy field or endoscopy narrative.

#### Report Classification

Classification of both endoscopy and pathology reports was performed in two stages: an automated preclassification using a custom Python script with regular expressions and keyword patterns covering affirmative, negated, suspicion-level, and differential-diagnostic phrasings, followed by complete manual review and correction by two reviewers. The analysis measured agreement with institutional histopathology reports (reference standard). Reliability of label extraction was assessed in a random sample by overlapping annotations, blinded to original labels but not the paired report, and examined by Cohen’s kappa with 95% confidence intervals.

Examiner identity was not retained during data extraction. Antrum-and-corpus sampling was defined as documented biopsy sampling from both regions. This definition does not establish adherence to the complete updated Sydney biopsy protocol. 12

### Statistical Methods

Analyses were performed at two analysis levels. *Patient-level analyses* were based on the first eligible EGD per patient (index examination; *n* = 9,852), used for prevalence, descriptive statistics, and regression. *Examination-level analyses* treated each eligible examination as an independent observation ( *n* = 11,381) and were used for concordance analyses.

Descriptive statistics are reported as counts and percentages for categorical variables and as median (IQR) for continuous variables. For regression analyses, age was categorized as <50, 50–75, and >75 years, reflecting strata used in ongoing screening evaluations (RISC-VAL, DRKS00035939); decade-based categories were used for visualization and exploratory analyses. Associations of age group and sex with each histologic outcome were modeled by multivariable logistic regression, with results reported as odds ratios (ORs) and 95% CIs (reference: <50 years, female sex).

For the concordance analysis, sensitivity, specificity, positive, and negative predictive values were calculated with 95% CIs by comparing endoscopic documentation to the histologic reference. Confidence intervals were calculated using the Clopper–Pearson method without adjustment for clustering within patients or endoscopists. Endoscopically undocumented cases were defined as histology-positive examinations without corresponding endoscopic documentation; overcalled cases as histology-negative examinations with positive endoscopic documentation. Site-specific analyses were performed for atrophy and IM. Two prespecified sensitivity analyses were performed: restriction to the patient-level, to address potential within-patient correlation, and restriction to antrum-and-corpus sampling examinations.

Documentation before (2016–2018) and after (2020–2024) publication of the MAPS II guideline 13 was compared by logistic regression within histology-positive examinations, adjusted for age group and sex, with 2019 excluded as transition year. For histologic prevalence, annual estimates were directly standardized to the age (decade) and sex distribution of the overall patient-level cohort, and calendar trends were modeled by logistic regression with calendar year as a continuous covariate, adjusted for age group and sex. The incomplete year 2025 was excluded from temporal regression models. The transition from EVIS EXERA III to EVIS X1 (Olympus) in 2021 is indicated. High-definition endoscopy with narrow-band imaging (Olympus 190 series) was available throughout the study period.

Analyses were performed on a complete-case basis; examinations with missing or inconclusive reports had been excluded during cohort identification ( Fig. 1 ). Analyses were performed in R (version 4.5.2); figures were created with ggplot2.

## Results

### Study Population

Between January 1, 2016 and March 31, 2025, a total of 42,698 upper gastrointestinal endoscopies in 28,561 patients were identified in the institutional endoscopy archive, of which 40,707 were diagnostic gastroscopies (jejunoscopies and therapeutic procedures without gastric biopsies could be identified from July 2022 onward only). After application of all exclusion criteria, 11,381 unique EGDs with gastric biopsies and conclusive histology from 9,852 patients were eligible for analysis, forming the examination-level and patient-level cohorts, respectively ( Fig. 1 ). Of the 9,852 patients, 1,121 (11.4%) contributed two or more examinations (two, *n* = 861; three, *n* = 179; four or more, *n* = 81; maximum, 10), accounting for 2,650 (23.3%) of all examinations.

### Cohort Characteristics and Overall Histologic Prevalence

Characteristics of the patient level cohort are shown in Table 1 . Median age was 66 years (IQR 52–77), and 52.4% were male. Antrum-and-corpus sampling was documented in 90.7% of index examinations.

**Table 1:** Characteristics of the patient-level cohort, overall and by age group.

Characteristic	Overall	<50 years	50–75 years	>75 years
**Demographics**				
Patients, *n*	9,852	2,092	4,936	2,824
Age, median [IQR], years	66.0 [52.0; 77.0]	37.0 [28.0; 44.0]	64.0 [58.0; 70.0]	81.0 [78.0; 84.0]
Male sex, *n* (%)	5,165 (52.4%)	1,029 (49.2%)	2,754 (55.8%)	1,382 (48.9%)
**Sampling**				
Antrum-and-corpus sampling, *n* (%)	8,931 (90.7%)	1,962 (93.8%)	4,443 (90.0%)	2,526 (89.4%)
**Histologic findings**				
*H. pylori* positive, *n* (%)	1,133 (11.5%)	325 (15.5%)	552 (11.2%)	256 (9.1%)
Atrophy positive, *n* (%)	654 (6.6%)	51 (2.4%)	318 (6.4%)	285 (10.1%)
Intestinal metaplasia positive, *n* (%)	2,119 (21.5%)	189 (9.0%)	1,080 (21.9%)	850 (30.1%)
*H. pylori* infection or any gastric precancerous condition *, n (%)*	3,096 (31.4%)	499 (23.9%)	1,534 (31.1%)	1,063 (37.6%)
**Extent and anatomical distribution**				
IM-positive examinations with anatomical assignment, *n*	2,089	189	1,061	839
Distal IM (antrum-only), *n* (%)	1,504 (72.0%)	146 (77.2%)	766 (72.2%)	592 (70.6%)
Extensive IM (corpus and/or fundus), *n* (%)	585 (28.0%)	43 (22.8%)	295 (27.8%)	247 (29.4%)
Atrophy-positive examinations with anatomical assignment, *n*	650	51	315	284
Reported antral involvement, *n* (%)	99 (15.2%)	5 (9.8%)	58 (18.4%)	36 (12.7%)
Reported corpus/fundus involvement, *n* (%)	580 (89.2%)	48 (94.1%)	271 (86.0%)	261 (91.9%)

Cohort characteristics are shown for the patient-level cohort ( *n* = 9,852), defined as the first eligible EGD with gastric biopsies per patient during the study period (January 2016–March 2025). Continuous variables are reported as median [IQR], categorical variables as counts and percentages. Distal IM was defined as antrum-only intestinal metaplasia; extensive IM as any IM involving the corpus and/or fundus, irrespective of additional antral involvement. The two IM categories are mutually exclusive. For atrophy, an analogous mutually exclusive classification was not applied due to histopathological underreporting. Twenty-nine index examinations had reported atrophy in both the antrum and corpus/fundus. IM = intestinal metaplasia; IQR = interquartile range.

Histologically confirmed *H. pylori* was present in 11.5% of index examinations, gastric atrophy in 6.6%, and IM in 21.5%. The composite outcome, “ *H. pylori* infection or any gastric precancerous condition,” was present in 31.4%. Prevalence estimates in the antrum-and-corpus subset ( *n* = 8,931) were within 0.5 percentage points of the overall cohort across all four outcomes ( **Supplementary Table 1** ).

Histologic prevalence patterns differed markedly across age groups. *H. pylori* prevalence decreased with increasing age, from 15.5% in patients younger than 50 years to 9.1% in those over 75 years. By contrast, atrophy increased from 2.4% to 10.1% and IM from 9.0% to 30.1% across the same age strata. The composite outcome “ *H. pylori* infection or any gastric precancerous condition” rose from 23.9% to 37.6%. Age-related patterns based on finer decade-based categories, as well as prevalence trends across the study period (2016–2025), are shown in Fig. 2 .

**Fig. 2:**
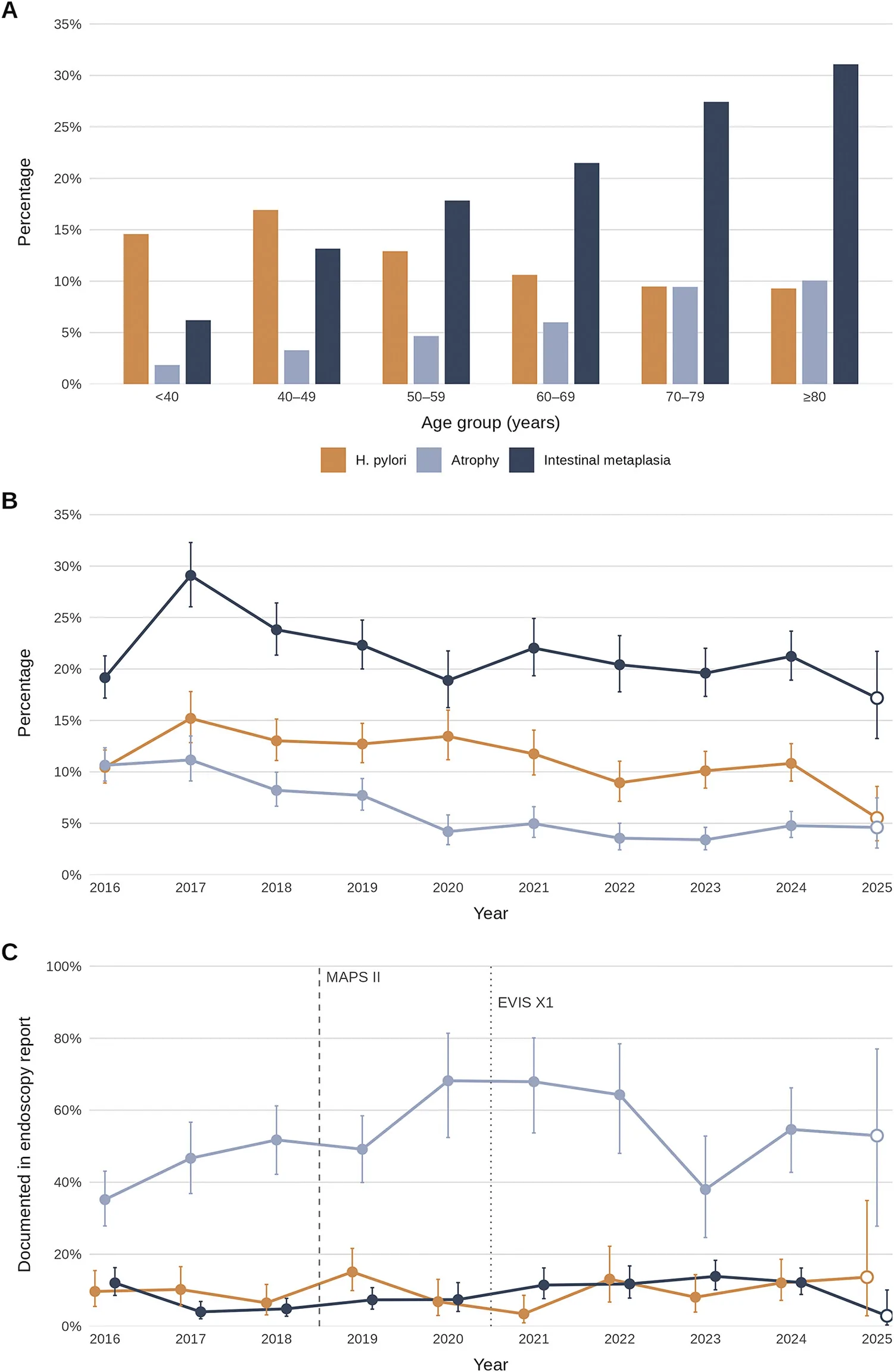
Histologic prevalence of *H. pylori* and gastric precancerous conditions. ( **A** ) Histologic prevalence by age decade and ( **B** ) annual histologic prevalence from 2016 to 2025 in the patient-level cohort ( *n* = 9,852). ( **C** ) Annual proportion of histology-positive examinations with corresponding endoscopic documentation in the examination-level cohort ( *n* = 11,381). The dashed and dotted lines mark publication of the MAPS II guideline (2019) and the introduction of EVIS X1 processors in our endoscopy unit (2021). Vertical bars represent 95% Clopper–Pearson confidence intervals. Data for 2025 cover only 1 January–31 March and are shown with open symbols.

### Temporal Trends in Documentation

Documentation of histologically confirmed *H. pylori* did not change after publication of MAPS II (8.7% in 2016–2018 vs. 8.6% in 2020–2024; adjusted OR 0.99, 95% CI 0.64–1.54; Fig. 2C ). Documentation of IM increased (6.9% vs. 11.6%; OR 1.82, 95% CI 1.35–2.49) but remained below 15% in every calendar year. For atrophy, documentation rose from 43.3% to 58.0% (OR 1.77, 95% CI 1.29–2.45). Over the same period, however, the histologic atrophy rate declined by approximately half, whereas the proportion of examinations with endoscopic atrophy statements remained stable. This increase largely therefore reflects a smaller histologic denominator rather than improved reporting. Results in the patient-level index cohort were consistent ( **Supplementary Table 2** ). Age and sex distribution remained stable across calendar years (median age 63–67 years; 50.7–56.9% male), and standardized prevalence differed from crude estimates by no more than 1 percentage point. After adjustment for age and sex, prevalence declined modestly for *H. pylori* (OR per year 0.97, 95% CI 0.95–0.99) and atrophy (OR 0.85, 95% CI 0.82–0.88; **Supplementary Table 3** ).

### Associations with Age Group and Sex

Associations differed by outcome ( Table 2 ). Compared with patients younger than 50 years, the odds of *H. pylori* were lower in those aged 50–75 (OR 0.68, 95% CI 0.58–0.79) and >75 years (OR 0.54, 0.45–0.65), whereas those of atrophy (OR 2.76 and 4.49) and IM (OR 2.78 and 4.35) increased. Male sex was associated with *H. pylori* (OR 1.18, 1.04–1.34) and IM (OR 1.25, 1.13–1.38), but not with atrophy (OR 0.99, 0.85–1.17).

**Table 2:** Multivariable association analyses for histologically confirmed *H. pylori* , atrophy, and intestinal metaplasia.

Outcome	*n* Positive	Variable	Level	Adjusted OR (95% CI)	Adjusted *p* -Value
*H. pylori*	1,133	Age group	50–75	0.68 (0.58–0.79)	<0.001
			>75	0.54 (0.45–0.65)	<0.001
		Sex	Male	1.18 (1.04–1.34)	0.009
Atrophy	654	Age group	50–75	2.76 (2.06–3.76)	<0.001
			>75	4.49 (3.35–6.15)	<0.001
		Sex	Male	0.99 (0.85–1.17)	0.936
Intestinal metaplasia	2,119	Age group	50–75	2.78 (2.37–3.29)	<0.001
			>75	4.35 (3.68–5.17)	<0.001
		Sex	Male	1.25 (1.13–1.38)	<0.001

Patient-level cohort ( *n* = 9,852). Adjusted odds ratios with 95% confidence intervals were derived from multivariable logistic regression models including age group and sex simultaneously, with <50 years and female sex as reference categories. CI, confidence interval; OR, odds ratio.

### Endoscopic Documentation of Histologically Confirmed Findings

Concordance between endoscopic documentation and the histologic reference is shown in Table 3 . Endoscopic documentation of histologically confirmed lesions was present in 9.6% of *H. pylori* cases (95% CI 8.0–11.4%), 9.2% of IM cases (95% CI 8.1–10.3%), and in 49.4% of atrophy cases (95% CI 45.8–52.9%). Positive predictive value was highest for IM at 73.2% (95% CI 68.1–78.0%), and lower for *H. pylori* (28.4%, 95% CI 24.0–33.0%) and atrophy (25.7%, 95% CI 23.5–28.0%).

**Table 3:** Concordance between routine endoscopic documentation and histopathology for *H. pylori* , atrophy, and intestinal metaplasia.

Outcome	Metric	% (95% CI)	*n* / *N*
***H. pylori***	Sensitivity ^a^	9.6 (8.0–11.4)	116/1,209
	Specificity ^b^	97.1 (96.8–97.4)	9,879/10,172
	PPV ^c^	28.4 (24.0–33.0)	116/409
	NPV ^d^	90.0 (89.5–90.6)	9,879/10,972
**Atrophy**	Sensitivity ^a^	49.4 (45.8–52.9)	386/782
	Specificity ^b^	89.5 (88.9–90.0)	9,481/10,599
	PPV ^c^	25.7 (23.5–28.0)	386/1,504
	NPV ^d^	96.0 (95.6–96.4)	9,481/9,877
**Intestinal metaplasia**	Sensitivity ^a^	9.2 (8.1–10.3)	238/2,596
	Specificity ^b^	99.0 (98.8–99.2)	8,698/8,785
	PPV ^c^	73.2 (68.1–78.0)	238/325
	NPV ^d^	78.7 (77.9–79.4)	8,698/11,056

Examination-level analysis ( *n* = 11,381). Documentation-positive denotes an explicit endoscopic diagnosis or written suspicion of the respective finding. The metrics describe concordance with institutional histopathology reports. Estimates are reported with 95% Clopper–Pearson confidence intervals without adjustment for clustering within patients or endoscopists. Atrophy concordance metrics are exploratory because institutional histopathology reports may underreport atrophy. *n/N* denotes the numerator and denominator for each metric. CI, confidence interval; NPV, negative predictive value; PPV, positive predictive value.

^a^ Documentation rate among histology-positive examinations.

^b^ Absence of documentation among histology-negative examinations.

^c^ Histologic positivity among documentation-positive examinations.

^d^ Histologic negativity among documentation-negative examinations.

Inter-rater agreement for label extraction was almost perfect for five of six labels (κ 0.94–0.99) and substantial for endoscopic documentation of IM (κ 0.78, 95% CI 0.61–0.96; **Supplementary Table 4** ).

Estimates restricted to the antrum-and-corpus subset and to the patient-level index cohort differed by no more than 0.7 and 2.6 percentage points, respectively, from the primary analysis across all entities and metrics ( **Supplementary Fig. 1** ). Adding RUT to the analysis increased documentation of *H. pylori* from 409 (3.6%) to 3,605 (31.7%) examinations overall and from 9.6% to 46.0% histology-positive examinations ( **Supplementary Table 5** ).

### Patterns of Endoscopic Underdocumentation and Apparent Overcalling

For *H. pylori* and IM, the low documentation rate reflected underdocumentation of histologically confirmed lesions rather than apparent overcalling of histologically negative ones; for atrophy, this pattern was reversed ( Fig. 3A ). Endoscopic documentation in the absence of histologic confirmation occurred in 2.9% of *H. pylori* -negative, 1.0% of IM-negative, and 10.5% of atrophy-negative examinations. In absolute numbers, undocumented cases outnumbered overcalled cases for *H. pylori* (1,093 vs. 293) and IM (2,358 vs. 87), whereas for atrophy overcalled cases predominated (1,118 vs. 396).

**Fig. 3:**
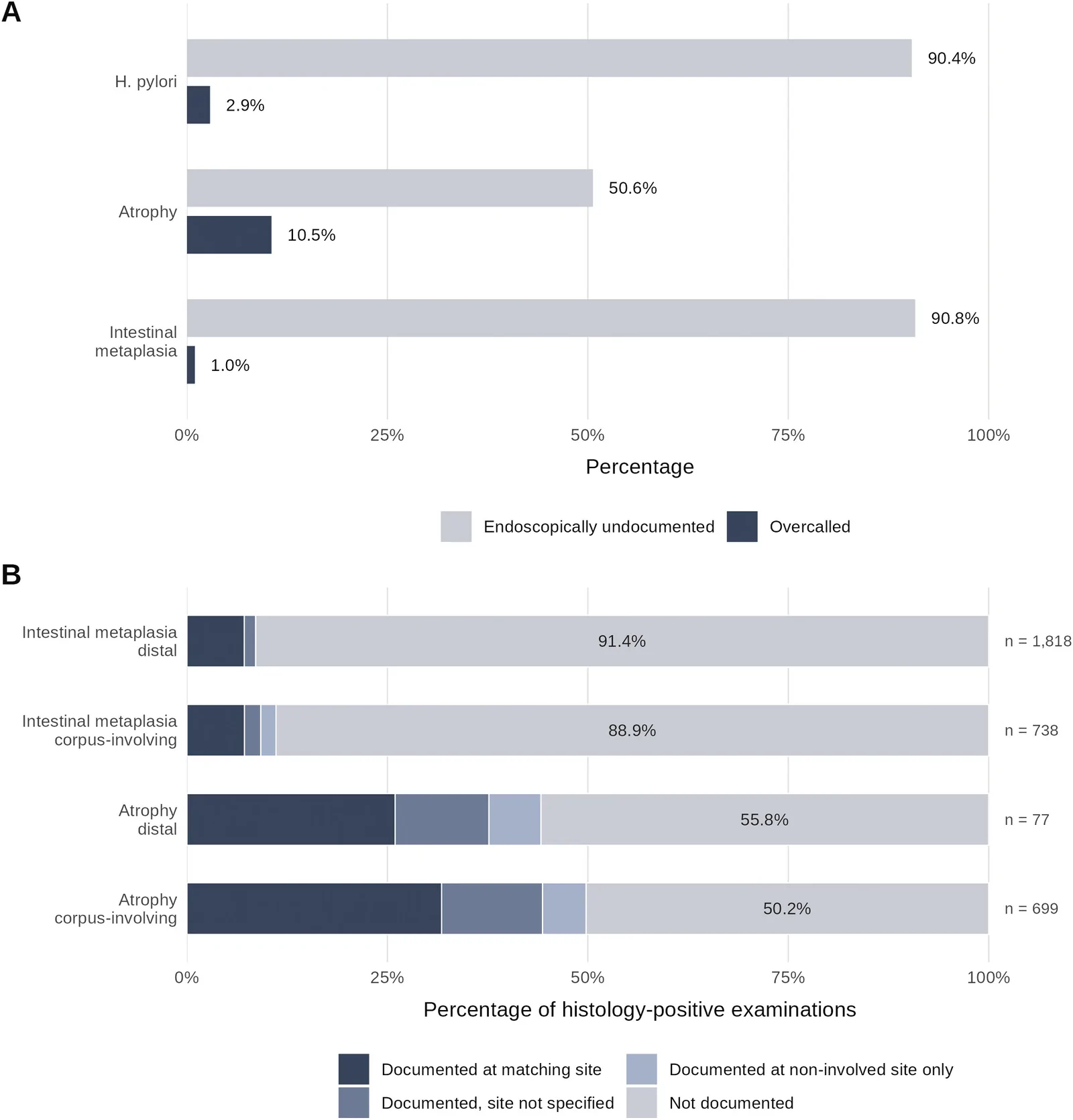
Endoscopic documentation of *H. pylori* , atrophy, and intestinal metaplasia. Analysis of the examination-level cohort. ( **A** ) Undocumented cases (light bars) are histology-positive examinations without corresponding endoscopic documentation; overcalled cases (dark bars) are histology-negative examinations with positive endoscopic documentation. “Overcalling” denotes discordance with histologic reporting, not necessarily a false-positive endoscopic finding. ( **B** ) Documentation of histology-positive examinations by extent. Distal = antrum only. Examinations without histologic site assignment are not shown (intestinal metaplasia, *n* = 40; atrophy, *n* = 6). Detailed estimates are given in the **Supplementary Table 6** .

Stratified by histologic extent, IM was documented in 8.6% of antrum-only and 11.1% of corpus-involving cases ( Fig. 3B ). Documentation at a histologically involved site was 7.2% in both groups. For atrophy, the corresponding proportions were 44.2% and 49.8% ( **Supplementary Table 6** ). Within antrum-and-corpus-sampling examinations, endoscopic statements of antral atrophy were confirmed at the antrum in 4.1% and statements of corpus atrophy in the corpus in 28.1%. For IM, the corresponding proportions were 70.6% and 33.3% ( **Supplementary Table 7** ).

### Histologic Extent and Reported Location of Lesions

Of the 2,119 index examinations with histologically confirmed IM, anatomical localization could be assigned to 2,089 (98.6%). Among classifiable examinations, distal IM (antrum-only) was more common than extensive IM (corpus and/or fundus; 72.0% vs. 28.0%; Table 1 ). For atrophy, reported corpus/fundus involvement (89.2%) substantially exceeded reported antral involvement (15.2%); categories were not mutually exclusive. The single-site anatomical breakdown for both lesions is shown in **Supplementary Fig. 2** . No explicit OLGA or OLGIM staging was identified in pathology reports in any calendar year.

## Discussion

This study delivers two key findings from routine clinical practice. First, gastric precancerous conditions and *H. pylori* infection are common in a contemporary German routine endoscopy cohort: nearly one in three patients undergoing a diagnostic EGD with biopsies harbored a histologically confirmed finding along the Correa cascade. Second, routine endoscopic reports mentioned only a small fraction of these lesions—written suspicion was below 10% of histologically confirmed *H. pylori* and IM cases and just under half of atrophy cases.

Our findings add contemporary German real-world data to a field dominated by selected cohorts and registry-linked studies. They are consistent with Dutch work by de Vries 14 showing that gastric precancerous lesions are clinically relevant in Western populations and confer substantial long-term cancer risk. These results are relevant to intensified European efforts toward structured gastric cancer screening (e.g., TOGAS). 15 Still, comparable contemporary German routine-cohort data are scarce. The ongoing HelicoPTER program was initiated specifically because current population-level *H. pylori* prevalence data in Germany are considered limited and outdated, with preliminary results suggesting lower infection rates than the historically cited figure of approximately one-third. 16 Our *H. pylori* prevalence of 11.5% is consistent with this trend, and prevalence in our cohort showed a modest decline across the study period that persisted after adjustment for age and sex (OR per year 0.97).

Birth-cohort effects generally predict higher *H. pylori* prevalence in older generations and do not readily explain the inverse age association observed here. 17 Lower histologic positivity in older patients may reflect prior eradication, reduced histologic detection during PPI use or in advanced atrophy, and selection of biopsied patients. Their relative contributions could not be determined. Conversely, atrophy and IM accumulate as long-term sequelae of prior infection, explaining their increase with age. IM may persist after *H. pylori* eradication, whereas our *H. pylori* estimate reflects histologic detection at the index examination. Hence, lower *H. pylori* prevalence does not contradict higher IM prevalence. The decline in histologic atrophy prevalence from 2020 onward persisted after age and sex standardization and was not paralleled by changes in sampling or endoscopic reporting.

The pronounced gap between histologic burden and routine endoscopic documentation has direct clinical implications, and is in line with recent European data, where endoscopic documentation rates of 48.5% for atrophy and 16.3% for IM were reported across three UK and Dutch centers. 18 The high PPV for IM (73.2%) indicates that when suggestive features were documented, they were usually confirmed histologically. For *H. pylori* , including RUT performance in the analysis increased the proportion of documentation from 9.6% to 46.0%. Thus, infrequent written suspicion should not be equated with absence of *H. pylori* testing or inadequate clinical assessment. The high specificity for *H. pylori* and IM is a function of how rarely these lesions were mentioned at all and therefore adds little beyond the low documentation rate itself. Limited use of structured descriptors, such as those of the Kyoto classification, may contribute to infrequent written suspicion. Their use was not assessed in this cohort. The absence of explicit OLGA/OLGIM staging also identifies an opportunity to improve structured reporting in histopathology.

Reliance on histopathology presupposes biopsy sampling. Gastric biopsies were obtained in only a subset of diagnostic gastroscopies ( Fig. 1 ), although the denominator was not equivalently cleaned, deduplicated or restricted to adults, precluding a reliable biopsy-rate estimate. Our data cannot determine whether biopsies were indicated in nonbiopsied examinations. Within the biopsied cohort, the documentation gap persisted in examinations with antrum-and-corpus sampling, underscoring the importance of recording suggestive mucosal findings even when histologic confirmation is anticipated.

Our results do not contradict prospective detection studies reporting substantially better performance under optimized conditions with narrow-band imaging or magnification; nor do they measure detection capability, since only biopsied examinations were analyzed. They quantify how completely findings in routine practice are recorded in the report. Documentation also did not improve substantially over time. Following publication of MAPS II in 2019, documentation of *H. pylori* remained unchanged and that of IM rose only modestly. Guideline availability alone has therefore not translated into more complete reporting, and neither did the transition to a new endoscope generation in 2021. High-definition and narrow-band imaging was available throughout, suggesting that technical capability was not the limiting factor, further supporting structured reporting as an active intervention.

The clinical relevance of these findings differs across the three lesions and across anatomical extent. *H. pylori* identification triggers eradication therapy, whereas surveillance for atrophy and IM depends on disease extent, severity and additional risk factors under MAPS III. 5 Antrum-restricted findings would in many cases not meet surveillance thresholds, whereas extensive antral IM including the incisura—histologically antral mucosa—may reach EGGIM 5 without corpus involvement. Failure to document advanced or extensive IM may therefore have greater implications for risk stratification and surveillance than failure to document limited antral IM without additional risk factors. Our data do not allow EGGIM or OLGA/OLGIM staging. However, histologic corpus involvement, as a proxy for the extensive atrophic gastritis that informs surveillance decisions under MAPS III, was not mentioned in half of the endoscopy reports (50.2%) and was attributed to the corpus/fundus in only 31.8%. IM involving both antrum and corpus remained undocumented in 82.5%.

A key strength is the scale of the linked endoscopy–histopathology cohort. For context, European studies of endoscopic–histologic agreement included 396 patients with histologically confirmed atrophy or IM 18 and 2,099 patients undergoing gastroscopy with biopsies for gastritis evaluation. 19 Additionally, we performed a deterministic two-stage label extraction, complete manual verification, institutional histopathological reference standard, and prespecified sensitivity analyses.

Several limitations warrant consideration. The single-center tertiary-care setting limits generalizability, and overrepresentation of symptomatic patients may inflate prevalence. Indication, proton pump inhibitor use, recent antibiotic exposure, prior eradication and region of origin were unavailable, so models were adjusted for age and sex only, and birth-cohort effects could not be separated. These factors, together with patchy infection and variable staining, may have reduced histologic detection of *H. pylori* and thus the measured PPV, to an extent that could not be assessed. Characteristics of nonbiopsied EGDs were unavailable, and restriction to biopsied examinations introduces selection and partial verification bias of undetermined magnitude and direction. Excluding therapeutic EGDs may additionally have lowered documentation rates. We assessed written documentation, not visual recognition. Coders were not blinded to the paired report, which would, if anything, favor concordant labeling. As antral atrophy is not routinely reported as a separate diagnosis at our institution, endoscopic statements of antral atrophy were almost never confirmed at the antrum (4.1%). Confirmation of corpus statements was also low (28.1%), which may reflect patchy distribution and limited sampling as well as overcalling. Atrophy concordance metrics are therefore exploratory, and “overcalling” does not necessarily indicate an endoscopic false positive. Examination-level confidence intervals were not adjusted for clustering within patients or endoscopists, whose identifiers were unavailable, and may be too narrow. Although 1,121 patients (11.4%) contributed more than one examination, the patient-level sensitivity analysis yielded consistent results. Prevalence estimates do not represent population prevalence, and p-values and confidence intervals should be interpreted descriptively, as no adjustment for multiple testing was made.

## Conclusion

In a large contemporary German routine cohort, *H. pylori* infection and gastric precancerous conditions were common among patients undergoing diagnostic gastroscopy with biopsies, yet were recorded only to a limited extent in the corresponding endoscopy reports. Within biopsied examinations, most already sampled both antrum and corpus, so closing this gap requires the reporting to change: explicit recording of suggestive mucosal patterns, standardized descriptors such as EGGIM, KTC, or the Kyoto-classification and structured or AI-assisted reporting. Without such changes, the established preventive potential of the Correa cascade will continue to be underutilized in routine clinical care.

## Data Availability

Data can be made available in respect to local regulatory affairs and on reasonable request.

## Declaration of GenAI Use

During revision of this manuscript, the authors used Claude Opus 5.5 (Anthropic) to assist with language editing and improve clarity. The authors reviewed and edited the resulting text and take full responsibility for the final manuscript.

## Supplementary Material

Ergänzendes Material Supplementary Material
