# Supplementary material for: Prevalence and Endoscopic Documentation of Helicobacter pylori and Gastric Precancerous Conditions in Routine Gastroscopy with Biopsies: A Cross-Sectional Study
Source: Endosc Int Open. 2026 Nov 6;14:a29921623. doi: 10.1055/a-2992-1623 (PMC13758972; doi:10.1055/a-2992-1623)
Supplement: Supplementary file 1 [file 10-1055-a-2992-1623_29934247.pdf]

Supplementary Material

Prevalence and endoscopic documentation of H. pylori and gastric precancerous conditions in routine gastroscopy with biopsies: a cross-sectional study

Roser DA, Ganthaler H, Probst A, Ignatenko Y, Freuer D, Messmann H, Römmele C

Supplementary Table 1 Histologic prevalence of H. pylori, atrophy, intestinal metaplasia, and the composite outcome in the patient-level analysis and in the antrum-and-corpus subset

Pre-specified sensitivity analysis comparing the overall patient-level cohort (*n* = 9,852) with the subset of patient-level examinations meeting the operational definition of antrum-and-corpus biopsy sampling (presence of biopsies from both antrum and corpus; *n* = 8,931). *H. pylori* or any gastric precancerous condition was defined as histologic evidence of *H. pylori*, atrophy, or intestinal metaplasia. Differences (in percentage points) are shown to facilitate direct comparison between cohorts.

Outcome	Overall index cohort, *n* (%)	Antrum-and-corpus subset, *n* (%)	Difference
H. pylori	1,133/9,852 (11.5%)	1,075/8,931 (12.0%)	+0.5 pp
Atrophy	654/9,852 (6.6%)	581/8,931 (6.5%)	-0.1 pp
Intestinal metaplasia	2,119/9,852 (21.5%)	1,877/8,931 (21.0%)	-0.5 pp
H. pylori infection or any gastric precancerous condition	3,096/9,852 (31.4%)	2,791/8,931 (31.3%)	-0.1 pp

Abbreviation: pp, percentage points.

Supplementary Table 2 Temporal trends in endoscopic documentation of histologically confirmed *H. pylori*, atrophy and intestinal metaplasia

Exploratory analysis of the proportion of histology-positive examinations with corresponding endoscopic documentation, comparing the examination-level cohort (*n* = 11,381) with the patient-level index examination cohort (*n* = 9,852). Adjusted odds ratios were derived from logistic regression models within histology-positive examinations, including period (2020–2024 vs. 2016–2018; 2019 excluded as year of publication of the MAPS II guideline and 2025 excluded as incompletely covered year) together with age group and sex.

Outcome	Analysis set (histology-positive, *n*)	Documented 2016–2018, *n/N* (%)	Documented 2020–2024, *n/N* (%)	Adjusted OR 2020–2024 vs. 2016–2018 (95% CI)	*p*-Value
H. pylori	Examination level (1,028)	39/446 (8.7%)	50/582 (8.6%)	0.99 (0.64–1.54)	0.960
	Patient-level index (960)	36/426 (8.5%)	44/534 (8.2%)	0.98 (0.62–1.56)	0.920
Atrophy	Examination level (645)	165/381 (43.3%)	153/264 (58.0%)	1.77 (1.29–2.45)	<0.001
